# 395. Real-World Insights of the Financial Metrics of a Long-Acting CAB/RPV Program

**DOI:** 10.1093/ofid/ofaf695.133

**Published:** 2026-01-11

**Authors:** Josh Havens, Jennifer O’Neill, Maureen Kubat, Sara H Bares, Nada Fadul, Jennifer M Davis, Josh Lechner, Shawnalyn Sunagawa

**Affiliations:** University of Nebraska Medical Center, Omaha, NE; University of Nebraska Medical Center, Omaha, NE; University of Nebraska Medical Center, Omaha, NE; University of Nebraska Medical Center, Omaha, NE; University of Nebraska Medical Center, Omaha, NE; University of Nebraska Medical Center, Omaha, NE; University of Nebraska Medical Center, Omaha, NE; University of Nebraska Medical Center, Omaha, NE

## Abstract

**Background:**

Utilization of long-acting cabotegravir/rilpivirine (CAB/RPV) for people with HIV (PWH) has expanded since FDA approval in 2021, but barriers remain. Its economic impact is unclear yet vital for equitable access and sustainability. We assessed the economic outcomes of CAB/RPV in a Midwestern US HIV clinic.

**Figure 1.**
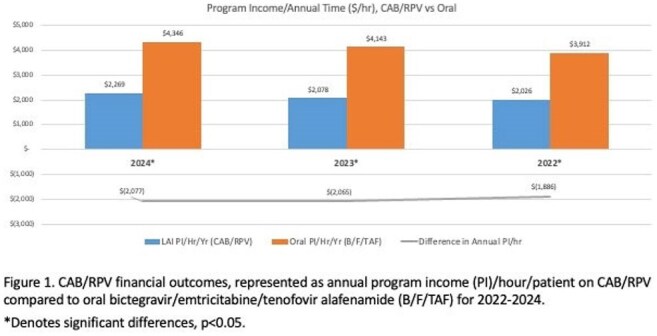
CAB/RPV financial outcomes. Represented as annual program income (PI)/hour/patient on CAB/RPV compared to oral bictegravir/emtricitabine/tenofovir alafenamide (B/F/TAF) for 2022-2024. *Denotes significant differences, p<0.05.

**Table 1.**
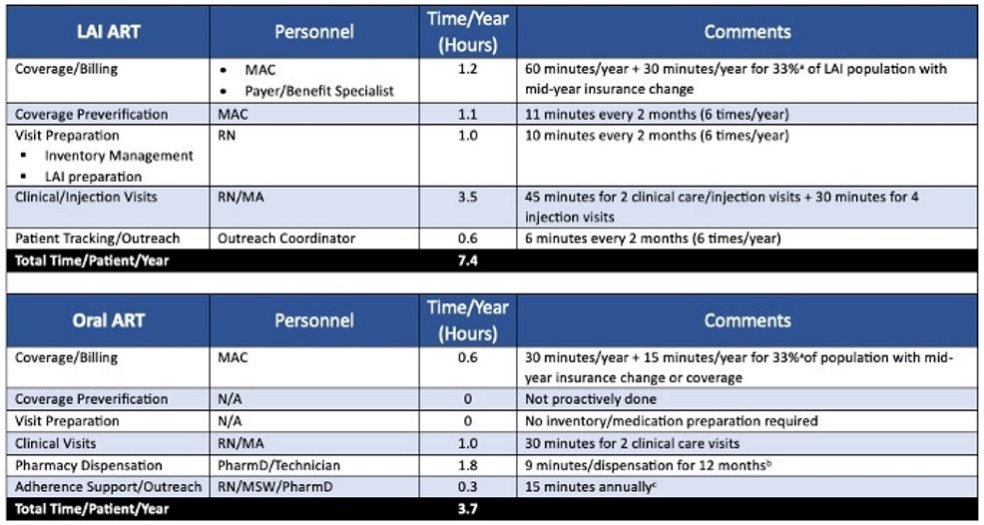
Time Estimates/LAI Patient Comparison of annual time estimates required to manage a single patient on long-acting injectable (LAI) antiretroviral therapy (ART) and oral ART. aBased on internal rates of insurance coverage formulation changes and/or switching coverage. bAssumption of perfect adherence with insurance requiring 30 day dispensations for a conservative viewpoint. Time required for 3 month dispensations would require less time (i.e. 0.6 hours annually). Definitions: MAC, medication access coordinator; RN, registered nurse; MA, medical assistant; PharmD, pharmacist; MSW, medical social worker.

**Methods:**

We conducted a retrospective cohort study of PWH on CAB/RPV (May 2022–December 2024). All CAB/RPV injections were accessed via buy-and-bill mechanisms. Demographics, clinical, and economic data were collected. Time studies estimated annual time required to manage long-acting inectable (LAI) and oral ART using bictegravir/emtricitabine/tenofovir alafenamide (B/F/TAF) as a comparator (Table 1). The primary outcome was annual program income (PI) per annual time spent for CAB/RPV vs. B/F/TAF (Wilcoxon Signed Rank). Secondary outcomes included mean PI and patient cost (PC) by year and payer type (Kruskal-Wallis).

**Table 2.**
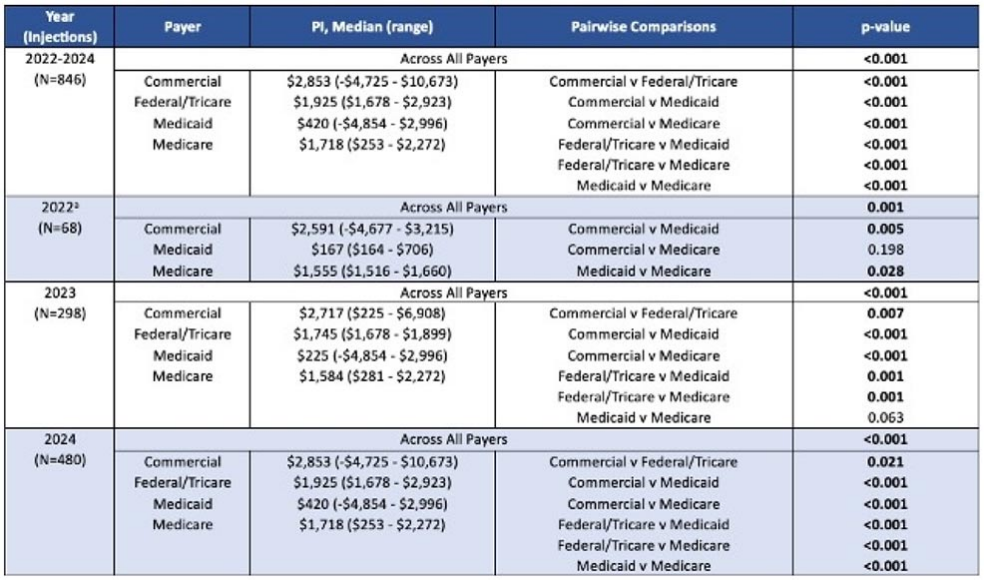
Comparison of median program income (PI) across study period years and payer groups. aNo patients had Federal/Tricare coverage during this year.

**Results:**

Within the study period, 113 patients received 846 CAB/RPV injections. Mean age was 46 years; 79% cisgender men, 18% Black, 13% Hispanic, and 11% Medicaid (MCD). Median (range) PI and PC per injection were $2,591 (-$4,854 - $10,673) and $0 ($0 - $1,324), respectively. Secondary coverage was used for 79% of injections (80% manufacturer-based; 5% ADAP). The majority of LAI injections (n=769, 91%) resulted in $0 PC for the patient. Negative PI occurred in 38 (4%) injections (median, -$2685; range, -$178 to -$4854); 92% covered by MCD and 76% occurring in 2024. Estimated annual time required to manage a patient on CAB/RPV and B/F/TAF was 7.4 and 3.7 hours, respectively. Annual PI/time was significantly less for CAB/RPV vs. B/F/TAF (p< 0.001) for each year (Fig. 1). Mean year-over-year (YoY) change in PI and PC per injection was 15% and -30%, respectively. PI significantly differed across payer types for all years (p< 0.001) (Table 2). CAB/RPV covered by MCD had lower PI than all other payer types.

**Conclusion:**

Over 2.5 years of operating a CAB/RPV LAI program, we found positive PI and low PC. Despite strong overall financial metrics, MCD-covered injections had the lowest and most negative PI, raising equity concerns. Ongoing evaluation of LAI financial outcomes is vital and needed for CAB/RPV sustainability.

**Disclosures:**

Josh Havens, PharmD, Gilead Biosciences: Grant/Research Support|GSK/ViiV Healthcare: Advisor/Consultant|Merck and Company, Inc.: Advisor/Consultant Sara H. Bares, MD, Gilead Sciences: Expert Testimony Jennifer M. Davis, MD, GSK/ViiV Healthcare: Grant/Research Support

